# 

**Published:** 1887-05-28

**Authors:** 


### THE HOSPITAL

An Institution, Family, and Parochial Journal of Hospitals, Asylums, & all Agencies for the Care of the Sick, Criticism & News.

### CONTENTS.

**Novel or No Novel?** - 135

**Words of Consolation.**—XXXV. The Work of the Holy Ghost- - 136

**Poetry.**—Somebody's Darling - 136

**Hospital Worthies.**—Messenger Monsey, M.D., F.R.S. - 137

**Public and Private Nursing Institutions.**—By a Member of the London Association of Nurses - 138

**The Story of the Hospitals.**—The London Hospitals - 139

**A National Pension Fund for Hospital Officials and Nurses.**—The Views of Officials, Sisters, Nurses, and Others - 141

**Notes and News.**—Hospital Items.—The Aberdeen Royal Infirmary.—Vacancies.—The Irving Dramatic Club.—Donations to Medical Charities, etc. - 142

**Annotations.**—The Hospitals Association.—The Hospitals Weeks.—Do Nothings - 145

**Chats about Medical Museums.**—By One of the Crowd.—No. VI. St. Bartholomew's Hospital- - 146

**The Out-Patient Department.**—By Sir Andrew Clark, Bart., M.D.; Dr. Collier; Mr. Timothy Holmes; and Colonel Montefiore - 147

**Incidents of Animal Life.**—Fooling with a Panther - 149

**Till the Doctor Comes.**—XIX. Treatment of Emergencies - 149

**The Editor's Letter Box.**—Grateful Patients - 150

**Words for the Wise** - 150

**The Children's Corner.**—Puzzles, Answers, etc. - 151

**Amusements with Profit** - 151

**In- and Out-Patients' Hospital Diary** - 152

